# Myopia prevalence among students in nine-year and independent compulsory education schools: a retrospective cross-sectional study

**DOI:** 10.1186/s12886-026-05333-3

**Published:** 2026-09-15

**Authors:** Hui Zhou, Baogang Xing, Shuang Tian, Weihua Chen, Chengming Yang, Zixuan Xiao

**Affiliations:** 1 Department of Ophthalmology, Southern University of Science and Technology Hospital, Shenzhen, Guangdong Province The People’s Republic of China; 2 Department of Ophthalmology, The Chinese University of Hong Kong Shenzhen Medical Centre, Shenzhen, Guangdong Province The People’s Republic of China

**Keywords:** Myopia, Educational transition, Compulsory education, Retrospective Cross-sectional study, Nanshan district

## Abstract

**Background:**

This study aims to investigate the prevalence of myopia during compulsory education in the Nanshan district of Shenzhen and the relationship between myopic prevalence and different types of educational transitions.

**Methods:**

Three different schools were purposively selected from the Nanshan district. One independent primary school and one independent secondary school together represented the discontinuous educational transition, and one nine-year school represented the continuous educational transition. The binomial logistic regression analysis was conducted to explore educational transitions associated with myopic prevalence.

**Results:**

Eight thousand ten students from two different educational transitions were involved in this retrospective cross-sectional study. The prevalence of myopia in the primary level (Grades 1–6) was 54.59%, while that in the secondary level (Grades 7–9) was 86.45%. The binomial logistic regression model revealed that the occurrence of myopia was significantly associated with females (OR = 1.60, 95% CI: 1.44–1.78; *P* < 0.001) and higher grade levels (*P* < 0.001). In exploratory analysis, students in the continuous educational transition showed lower odds of myopia than those in the discontinuous educational transition (OR = 0.85, 95% CI: 0.77–0.95; *P* = 0.003).

**Conclusion:**

The prevalence of myopia was relatively high and surged from low-grade to high-grade levels among compulsory students in the Nanshan district. An association between the educational transition model and myopia prevalence was observed. However, this association was exploratory, because the educational transition exposure is fully confounded with school identity. Further longitudinal studies with multiple schools across diverse regions are warranted to clarify the specific mechanisms through which educational structure influences ocular health.

**Supplementary Information:**

The online version contains supplementary material available at https://doi.org/10.1186/s12886-026-05333-3.

## Background

Myopia, a refractive error that means the light rays from far distant objects focus in front of the retina, resulting in blurred vision, has become a global public health issue [1]. Previous studies estimated that the prevalence of myopia would explode in the next decades [2, 3]. The myopic complications are caused by an increase in the axial length and result in vision impairment [3–6]. The vision impairment caused by myopia not only impacts the daily life of patients but also increases the socioeconomic cost [1, 7, 8]. According to previous studies, the prevalence of myopia among primary and secondary school students in China is the highest in the world [3, 9]. Consequently, the Chinese government has promoted the “Double Reduction” policy, which alleviates educational stress and regular school vision screenings to mitigate the growing prevalence of myopia in children [10, 11].

Previous studies show that the development of myopia is affected by many factors, including genetic factors and environmental factors [12–15]. The nine-year compulsory education policy has been implemented nationwide in China, but there are different types of educational transition from the primary level (Grades 1–6) to the secondary level (Grades 7–9) [16]. “Nine-year school” is a kind of educational transition in which nine-year compulsory education is continuous, systemic, and unitary, while the independent school separates the primary level from the secondary level [17, 18]. Independent primary schools, independent secondary schools, and nine-year schools have different educational environments and different learning pressures [16]. The nuances caused by different educational transitions may affect the eye-use health behaviors of students.

To our knowledge, no study has investigated which kind of educational transitions during the compulsory education stage in China provide benefits for student vision health. Therefore, this study aims to compare the prevalence of myopia in schools with different educational transitions and to explore educational transitions associated with the prevalence of myopia.

## Methods

### Subjects and methods

This retrospective cross-sectional study was a secondary analysis of data from the routine annual school vision screening program conducted in May 2025. Three schools (one independent primary school, one independent secondary school, and one nine-year school) were purposively selected from the same Sub-District in the Nanshan district of Shenzhen to represent the two different educational transition types (discontinuous educational transition vs. continuous educational transition). This geographically proximate selection was intended to minimize socioeconomic confounding across schools. These schools are a subset of the schools included in the routine annual school vision screening program. This study has been approved by the Human Medical Ethics Committee of Southern University of Science and Technology Hospital (Approval No.2026-004, dated March 2, 2026), which was in accordance with the tenets of the Declaration of Helsinki. The requirement for individual consent was waived by the committee because of the retrospective nature of the study.

### Subjects

A total of 8,485 students from three schools were eligible for participation. 8,010 students (94.40%) completed the examination and were included in the analysis. The remaining 475 students (5.60%) did not participate in the project owing to absence on the examination day, physical discomfort, or inability to cooperate. Non-participants did not differ significantly from included students with respect to sex (χ²=1.257, *P* = 0.262) or age (non-participants: 10.77 ± 2.52 years vs. participants: 10.99 ± 2.59; U = 1,809,354, *P* = 0.071). Unfortunately, the grade level information of non-participants was unavailable. All participants received the visual acuity test (EV 1603-01, EyeVision Inc., Guangzhou, China) and non-cycloplegic autorefraction (AR-1, NIDEK, Tokyo, Japan) by a professional and trained visual screening team. Students wearing contact lenses were instructed to remove them at least 30 min before the refraction test. All the inspection procedures and sites complied with national standards [19]. Each subject underwent three-times non-cycloplegic autorefractions. These participants, whose differences among the three non-cycloplegic refraction results are greater than 0.25 Diopter(D) would be measured again to ensure that the data meet the standards.

According to the Chinese national screening standard (WS/T 663–2020), myopia was defined as uncorrected visual acuity of any eye < 5.0(Standard for logarithmic visual acuity charts; equivalent to Snellen acuity < 20/20 or logMAR > 0) with the spherical equivalent (SE) <-0.50 D (The SE was calculated as SE = sphere + 0.5*cylinder), or those who had a history of wearing orthokeratology lenses [19].

### Statistical analysis

The independent primary school and the independent secondary school were combined into the discontinuous educational transition, while a nine-year school was considered the continuous educational transition. All subjects were categorized into the myopia group and the no-myopia group according to the definition of myopia. Statistical analysis was performed with a commercially available software (SPSS ver. 26.0; SPSS Inc., Chicago, IL). The Shapiro-Wilk test was used to check the data distribution for normality. According to the data normality, the t-test or the Mann-Whitney U test was used to compare the age in two different educational transitions. The t-test or the Mann-Whitney U test was also used to compare the age between two study groups (myopia vs. non-myopia). The chi-squared test (χ² test) was used to compare the gender, grade level, and prevalence of myopia between two educational transitions. The χ² test was also used to compare the gender, grade level, and educational transition model between two study groups (myopia vs. non-myopia). The binomial logistic regression analysis was conducted to examine the impact of grade level, gender, and the educational transition on myopia. Because only three schools were included in the ordinary logistic regression, standard errors may be underestimated and the significance may be overstated. The variance inflation factor (VIF) diagnostic method was used to examine the multicollinearity of all predictor variables. Because there was a high collinearity between age and grade level, age was removed from the model. The VIF values of the rest of the variables were below 5, indicating no significant multicollinearity among the predictor variables. Additionally, to determine whether the association persisted within primary grades alone, a sensitivity analysis restricted to primary grades (Grades 1–6) was performed. *P* < 0.05 was considered as statistically significant. A sensitivity analysis based on spherical equivalent without the visual acuity criterion as an alternative myopia definition was also performed (Supplementary Table 1).

## Results

### Demographic characteristics and myopic status of students in different educational transitions

**Fig. 1:**
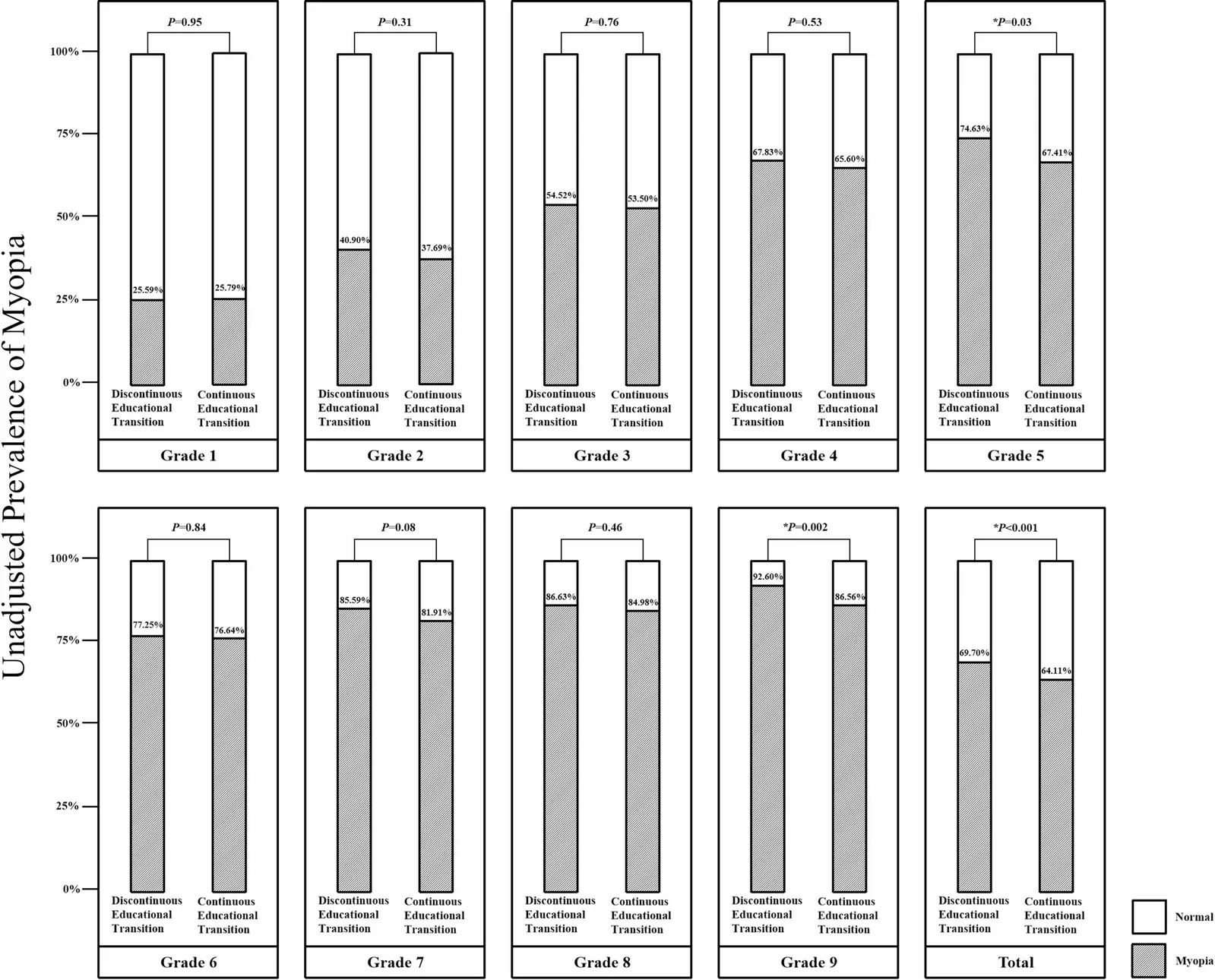
The unadjusted prevalence of myopia in different educational transitions according to grade. The chi-squared test (χ² test) was used to compare the unadjusted prevalence of myopia between two educational transitions. And the P values were not adjusted for sex or for differences in grade composition between two educational transitions

The prevalence of myopia in the primary level was 54.59%, while that in the secondary level was 86.45%. The demographic characteristics of different educational transitions in compulsory education are shown in Table 1. The mean ages of the discontinuous educational transition and continuous educational transition were 11.04 ± 2.48 years and 10.92 ± 2.72 years, respectively. There was a significant difference in the grade level between the discontinuous and continuous educational transition (χ²=50.04, *P* < 0.001). Specifically, the discontinuous educational transition was primarily composed of higher grade level, while the continuous educational transition was primarily composed of lower grade level. The prevalence of myopia among males and females in the discontinuous educational transition was higher than that in the continuous educational transition (Male: χ²=7.38, *P* = 0.007; Female: χ²=22.35, *P* < 0.001). The unadjusted prevalence of myopia in the continuous educational transition was significantly lower than in the discontinuous educational transition model (64.11% vs. 69.70%; χ²=27.95, *P* < 0.001). An upward trend of myopia with grade advancement was observed. The myopic prevalence of different educational transitions is shown in Fig. 1. There were no statistically significant differences in age and gender between different educational transitions. 

**Table 1:** The demographic characteristics of different educational transitions

	Discontinuous Educational Transition(*n* = 4485)	Continuous Educational Transition(*n* = 3525)	*P*
**Age (years, mean ± SD)**	11.04 ± 2.48	10.92 ± 2.72	0.06*
**Gender**,** n (%)**			0.09†
Male	2338(52.13%)	1904 (54.01%)	
Female	2147(47.87%)	1621(45.99%)	
**Grade level**,** n (%)**			< 0.001†
Grade 1	379(8.45%)	411(11.66%)	
Grade 2	533(11.88%)	451(12.79%)	
Grade 3	431(9.61%)	400(11.35%)	
Grade 4	429(9.57%)	332(9.42%)	
Grade 5	410(9.14%)	310(8.79%)	
Grade 6	422(9.41%)	321(9.11%)	
Grade 7	763(17.01%)	470(13.33%)	
Grade 8	591(13.18%)	406(11.52%)	
Grade 9	527(11.75%)	424(12.03%)	

n, number

SD, standard deviation

* Mann-Whitney U test. †χ^2^ test

### Myopia patterns among school children in compulsory education

The demographic characteristics of myopic students in compulsory education are shown in Table 2. The mean ages of the non-myopia group and myopia group were 9.42 ± 2.37 years and 11.75 ± 2.34 years, respectively. The students with myopia were significantly older than the students without myopia (U = 3452187.00, *P* < 0.001). There was a statistically significant difference in gender and grade level between the two groups (Gender: χ²=59.97, *P* < 0.001; Grade level: χ²=1609.44, *P* < 0.001). The non-myopia group was primarily composed of males (1 552/2 624, 59.15%), while the myopia group had a balanced gender distribution (Males: 2 690/5 386, 49.94%; Females: 2 696/5 386, 50.06%). The non-myopia group was primarily composed of lower-grade-level students (Grade 1–3), while the myopia group was primarily composed of higher-grade-level students (Grade 7–9). The statistically significant difference in educational model between the two study groups was observed (χ²=27.95, *P* < 0.001).

The binomial logistic regression model is shown in Table 3. McFadden’s pseudo-R² was 0.169 (χ² = 1714.08, *P* < 0.001), indicating a meaningful improvement existed in the model over the null model. The binomial logistic regression model revealed that the occurrence of myopia was significantly associated with females (OR = 1.60, 95% CI: 1.44–1.78; *P* < 0.001) and higher grade level (the largest effect observed at Grade 9, OR = 26.56, 95% CI: 20.36–34.66; *P* < 0.001). The students in the continuous transition were less likely to have myopia (OR = 0.85, 95% CI: 0.77–0.95; *P* = 0.003). However, when the analysis was restricted to primary grade level (Grade 1–6), the adjusted odds ratio for continuous transition decreased to 0.90 (95% CI: 0.80–1.02, *P* = 0.09), suggesting that the overall association was largely driven by secondary-grade composition rather than a uniform structural effect. The sensitivity analysis based on spherical equivalent without the visual acuity criterion had directionally consistent results, with an adjusted OR of 0.87 (95% CI: 0.78–0.96, *P* = 0.007) for continuous educational transition (Supplementary Table 1).

**Table 2:** The demographic characteristics of myopic students

	Non-myopia (*n* = 2624)	Myopia (*n* = 5386)	*P*
**Age (years, mean ± SD)**	9.42 ± 2.37	11.75 ± 2.34	< 0.001*
**Gender**,** n (%)**			< 0.001 †
Male	1552(59.15%)	2690(49.94%)	
Female	1072(40.85%)	2696(50.06%)	
**Grade level**,** n (%)**			< 0.001 †
Grade 1	587(22.37%)	203(3.77%)	
Grade 2	596(22.71%)	388(7.20%)	
Grade 3	382(14.56%)	449(8.34%)	
Grade 4	252(9.6%)	509(9.45%)	
Grade 5	205(7.81%)	515(9.56%)	
Grade 6	171(6.52%)	572(10.62%)	
Grade 7	195(7.43%)	1038(19.27%)	
Grade 8	140(5.34%)	857(15.91%)	
Grade 9	96(3.66%)	855(15.87%)	
**Educational transition model**,** n (%)**			< 0.001†
Discontinuous Educational Transition	1359(51.79%)	3126(58.04%)	
Continuous Educational Transition	1265(48.21%)	2260(41.96%)	

n, number

SD, standard deviation

* Mann-Whitney U test. †χ^2^ test

Percentages may not sum to 100% due to rounding

**Table 3:** Binomial logistic regression model of contributing factors to myopia

	OR (95% CI)	*P*
**Intercept**	0.30(0.25–0.36)	< 0.001
**Gender**		
Male	Reference	-
Female	1.60(1.44–1.78)	< 0.001
**Grade level**		
Grade 1	Reference	-
Grade 2	1.86(1.52–2.29)	< 0.001
Grade 3	3.45(2.79–4.26)	< 0.001
Grade 4	5.93(4.75–7.40)	< 0.001
Grade 5	7.27(5.78–9.14)	< 0.001
Grade 6	9.75(7.70–12.33)	< 0.001
Grade 7	15.40(12.33–19.24)	< 0.001
Grade 8	18.19(14.29–23.16)	< 0.001
Grade 9	26.56(20.36–34.66)	< 0.001
**Educational transition model**		
Discontinuous Educational Transition	Reference	-
Continuous Educational Transition	0.85(0.77–0.95)*	0.003

n, number

McFadden’s pseudo-R² =0.169

χ^2^ = 1714.08, *p* < 0.001

OR, odds ratio

CI, confidence interval

*The educational transition exposure is fully confounded with school identity (the continuous educational transition comprises a single nine-year school; the discontinuous educational transition comprises one independent primary and one independent secondary school). The transition OR is exploratory and cannot be separated from unmeasured school-level effects

## Discussion

This cross-sectional study investigated the prevalence of myopia in compulsory education in the Nanshan district of Shenzhen, and compared the prevalence of myopia in different educational transitions. The prevalence of myopia in the primary level exhibited a low level, while that in the secondary level increased. The female and higher grade level were possible associated factors for the occurrence of myopia, while the continuous transition in compulsory education was associated with lower odds of myopia in the adjusted model.

Previous studies have found that myopia is affected by genetic and environmental factors [12–15]. Recently, more and more studies have highlighted that environmental factors cannot be ignored [9, 20, 21]. With the development of the economy and informatization, more and more teenagers are exposed to electronic display terminal equipment prematurely and frequently, which leads to myopia in teenagers [10, 11, 22–24]. Myopia and its complications are a growing global health issue [2, 3]. The previous study in 2020 indicated that the prevalence of myopia among primary and secondary school students in Shenzhen was 50.50%, up 3.6% points from 2019 [9]. Another recent study found that the prevalence of myopia in Nanning City of Guangxi Province was 50.54% in primary school and 70.83% in secondary school [25]. The current study found that the prevalence of myopia in the Nanshan district was at a high level. Firstly, the data of this research is the latest, which is in line with the trend of myopia rate increase in the previous regional study in Shenzhen [9]; Secondly, the prevalence of myopia was proven to be associated with urbanization [9, 26]. The Nanshan district of Shenzhen is the birthplace of China’s reform and opening up, with a developed regional economy, high-quality educational resources, and a high level of urbanization. The students in highly urbanized areas tend to spend more time on different kinds of electronic products, which consequently reduces their rest time [9, 27].

This study found similar results to the prior studies, which indicated that increasing grade level was a possible associated factor for myopia [9, 12, 21, 28–32]. The increasing educational stress pushes students at high grade level to spend more time reading and writing [21, 33, 34]. The current study revealed that Grade 6 students exhibited the highest odds of myopia compared to all other grades in the primary level, while the Grade 9 students showed the highest odds of myopia within the secondary level. This pattern may coincide with the timing of China’s junior and senior secondary education entrance examinations, highlighting the significant role of intense educational stress in visual health degradation [12].

Moreover, this study indicated that the continuous educational transition was associated with lower odds of myopia (OR = 0.85, 95% CI: 0.77–0.95; *P* = 0.003). The students in the continuous educational transition received nine years of compulsory education at the same school without the stress of level transition, while the students in the discontinuous educational transition had to study in a different school during different stages [17]. The policy requiring primary school students to be admitted into secondary school without an entrance examination and attend a local school was first piloted in 2014 by the Chinese government [35]. Theoretically, there was no academic pressure during the transition from the primary level to the secondary level. However, this policy of degree only guarantees students’ basic admission qualifications. Although the quality of public educational resources was excellent, these excellent resources were insufficient in quantity [16]. Potential educational stress in independent primary schools persists [17, 18]. A nine-year school operating as a continuous educational transition in compulsory education may further alleviate the educational stress caused by competitive school admissions [17, 18]. Meanwhile, the nine-year school ensures coherence in its governance structures and institutional culture, while providing students with a stable environment for living and learning [17, 18]. The stable environment can reduce the students’ role-transition adaptation period, thereby benefiting their physical and psychological health [18]. However, the educational transition cannot be separated from school identity due to the design of the current study. If the association were driven by the educational transition structure, plausible mechanisms could include reduced stress from school-transition examinations [17, 18, 35] and greater stability in governance and institutional culture [17, 18], which may in turn promote healthier eye-use behaviors. Moreover, the analysis restricted to primary grade level (Grade 1–6) indicated that the association does not operate uniformly across grades. Combined with the confounding of school identity, these findings remain exploratory in the current study. Further studies with more multiple type schools are warranted to indicate the influence of educational structure.

This study found that female students tend to have more myopia than males, which was similar to prior studies [36–39]. Zhang et al. study indicated that the occurrence of myopia in women may be related to the early initiation of female puberty, and the increase of estrogen level in female puberty may promote the development of myopia by physiological mechanisms such as scleral remodeling [38].

There were several limitations in this cross-sectional exploratory study. First and most importantly, the educational transition exposure is fully confounded with school identity. The continuous educational transition consisted of a single nine-year school, while the discontinuous educational transition comprised one independent primary school and one independent secondary school. Different types of schools have different school-level variables, such as different class schedules and outdoor activity facilities; These school-level variables should not be ignored. However, these school-level variables cannot be separated from the educational transition model because of the limited research resources in the current study. Consequently, the observed educational transition odds ratio of 0.85 may reflect the specific characteristics of these three schools rather than an effect of the educational transition structure per se. This limitation precludes causal attribution. Moreover, there were many factors associated with myopia, including outdoor activity time, sleep duration, bedtime, nutritional intake, indoor lighting intensity, parental myopia, etc [9, 29–32, 40, 41]. These omitted factors may limit the model’s explanatory capacity; nevertheless, the model demonstrated a meaningful improvement in fit over the null model (McFadden’s pseudo-R² = 0.169, χ² = 1714.08, *P* < 0.001). There is a need for more specific studies that explore the educational structure that influences the development of myopia. Secondly, the effect of lens accommodation was not considered in the current study. The human eye can see near objects clearly by adjusting the degree of curvature of the convex lens. Children have a high accommodative amplitude, which makes non-cycloplegic refraction unreliable [42]. Measurement of refractive error without cycloplegia could overestimate the myopic status in children, especially for the students in lower grades [43, 44]. Although all the subjects underwent three-times non-cycloplegic autorefraction to make sure the accuracy of the data, non-cycloplegic autorefraction may lead to inaccurate results which overestimate myopia prevalence in lower grades than that in higher grades. These inaccurate results may conceal the true myopic prevalence surge from the primary educational stage to the secondary educational stage, which may be larger than the status reported in the current study. Future studies with the objective cycloplegic refraction were warranted to make data more accurate. A sensitivity analysis based on spherical equivalent without the visual acuity criterion as an alternative myopia definition yielded results consistent with the main findings (Supplementary Table 1), suggesting that the composite definition did not materially alter the study conclusions. Thirdly, we had to remove the age from the binomial logistic regression model, because there was a high degree of collinearity between age and grade level (the VIF = 35.847). The grade level can represent the level of academic stress better [12]. Finally, this study only assessed three schools from the same sub-district in the Nanshan district. Different childhood socioeconomic status was significantly associated with myopia [45, 46]. To minimize socioeconomic confounding, we used a geographic proximate strategy to select all participating schools. This sampling method ensured relative homogeneity in socioeconomic status among the student populations, and better isolated the effect of different types of schools on the myopic prevalence without the interference of drastic socioeconomic status disparities. However, the sampling design in the current study may underestimate standard errors and overstate the significance of logistic regression, because within-school correlation could not be modeled with only three schools. Data from this study cannot be extrapolated to the rural or smaller cities of China, and further research is needed in other regions. Because of the cross-sectional design, the causal relationship between the associations could not be established. Further longitudinal studies with more comprehensive measurements and optimized methods across diverse regions are warranted to clarify the specific mechanisms through which educational structure influences ocular health.

## Conclusion

The prevalence of myopia among students in compulsory education exhibited a relatively high level in the Nanshan district of Shenzhen, with rates escalating dramatically from primary to secondary level. The substantial increase in myopic prevalence across grade levels is consistent with national trends. The association between the low occurrence of myopia and the continuous educational transition was observed in this cross-sectional study (OR = 0.85, *P* = 0.003). However, this association was exploratory, because the educational transition exposure is fully confounded with school identity. It is noteworthy that before any consideration of educational structure in public health strategies for myopia prevention, further longitudinal studies with multiple schools across diverse regions are warranted.

## Supplementary Information

Below is the link to the electronic supplementary material.

Supplementary Material 1

## Data Availability

The raw data supporting the conclusions of this article will be made available by the corresponding author on reasonable request, without undue reservation.

## Abbreviations

n: number
SD: Standard deviation
SE: Spherical equivalent
OR: Odds ratio
CI: Confidence interval
