# Supplementary material for: Myopia prevalence among students in nine-year and independent compulsory education schools: a retrospective cross-sectional study
Source: BMC Ophthalmol. 2026 Sep 15;26:697. doi: 10.1186/s12886-026-05333-3 (PMC13718145; doi:10.1186/s12886-026-05333-3)
Supplement: Supplementary file 1 [file 12886_2026_5333_MOESM1_ESM.docx]

Supplementary Table 1. Sensitivity analysis: comparison of myopia prevalence and associated factors using the composite myopic definition vs. myopic definition without the visual acuity criterion.

|  | **Composite myopic definition**  **(main analysis)** | **Myopic definition without the visual acuity criterion**  **(sensitivity)** |
| --- | --- | --- |
| **Myopia prevalence, n (%)** | 5386(67.24%) | 5496(68.61%) |
| **Myopia prevalence in different educational transition model, n (%)** |  |  |
| Discontinuous Educational Transition | 3126(69.70%) | 3181(70.93%) |
| Continuous Educational Transition | 2260(64.11%) | 2315(65.67%) |
| **Myopia prevalence in different Grade level, n (%)** |  |  |
| Grade 1 | 203(25.70%) | 224(28.35%) |
| Grade 2 | 388(39.43%) | 417(42.38%) |
| Grade 3 | 449(54.03%) | 462(55.60%) |
| Grade 4 | 509(66.89%) | 516(67.81%) |
| Grade 5 | 515(71.53%) | 524(72.78%) |
| Grade 6 | 572(76.99%) | 582(78.33%) |
| Grade 7 | 1038(84.18%) | 1051(85.24%) |
| Grade 8 | 857(85.96%) | 860(86.26%) |
| Grade 9 | 855(89.91%) | 860(90.43%) |
| **Logistic regression (adjusted OR, 95% CI, P) *** |  |  |
| McFadden R² | 0.169 | 0.163 |
| Female (vs. Male) | 1.60(1.44–1.78), <0.001 | 1.61(1.44–1.79)， <0.001 |
| Grade 2(vs. Grade 1) | 1.86(1.52–2.29), <0.001 | 1.84(1.51–2.25), <0.001 |
| Grade 3(vs. Grade 1) | 3.45(2.79–4.26), <0.001 | 3.21(2.61–3.95), <0.001 |
| Grade 4(vs. Grade 1) | 5.93(4.75–7.40), <0.001 | 5.40(4.34–6.72), <0.001 |
| Grade 5(vs. Grade 1) | 7.27(5.78–9.14), <0.001 | 6.76(5.39–8.49), <0.001 |
| Grade 6(vs. Grade 1) | 9.75(7.70–12.33), <0.001 | 9.21(7.28–11.64), <0.001 |
| Grade 7(vs. Grade 1) | 15.40(12.33–19.24), <0.001 | 14.61(11.70–18.25), <0.001 |
| Grade 8(vs. Grade 1) | 18.19(14.29–23.16), <0.001 | 16.30(12.83–20.71), <0.001 |
| Grade 9(vs. Grade 1) | 26.56(20.36–34.66), <0.001 | 24.62(18.85–32.16), <0.001 |
| Continuous transition (vs. Discontinuous) | 0.85(0.77–0.95), 0.003 | 0.87(0.78–0.96)， 0.007 |

***n****, number*

***OR****, odds ratio.*

***CI****,* *confidence interval*

***Composite myopic definition:*** *uncorrected visual acuity < 5.0 (Standard for logarithmic visual acuity charts; equivalent to Snellen acuity < 20/20 or logMAR > 0) and SE < −0.50 D, or orthokeratology lens wear history.*

***Myopic definition without the visual acuity criterion:*** *SE < −0.50 D, or orthokeratology lens wear history.*

**All logistic regression models adjusted for gender, grade level, and educational transition model.*
